# Experimental study and machine learning modeling of water removal efficiency from crude oil using demulsifier

**DOI:** 10.1038/s41598-024-59963-y

**Published:** 2024-04-22

**Authors:** H. H. Hashem, T. Kikhavani, M. A. Moradkhani

**Affiliations:** Department of Chemical Engineering, Faculty of Engineering, Ilam University, Ilam, Iran

**Keywords:** Demulsifier, Crude oil, Water removal efficiency, Machine learning, Engineering, Mathematics and computing

## Abstract

This study deals with the investigation of the water removal efficiency (WRE) from crude oil using a commercial demulsifier. The impacts of time, demulsifier concentration, and temperature on WRE were experimentally studied. The results implied the fact that temperature plays a substantial role in the demulsification and has a direct correlation with WRE. In addition, while increasing the concentration up to 40 ppm contributed to reaching a higher WRE, it did not have positive effects on efficiency at higher concentrations (overdose) and just led to more demulsifier consumption. The concentration dependence of WRE was also diminished at high temperatures. At higher levels of temperature and concentration, the time required to reach a high WRE was noticeably reduced. In order to generalize the findings of this study, the measured experimental data were employed to design predictive methods for WRE based on two smart soft-computing paradigms, including Multilayer perceptron (MLP) and Gaussian process regression (GPR). Despite the high accuracy of both models, the MLP model presented the best consistencies with experimental data with average absolute relative error and relative root mean squared error of 0.84%, and 0.01%, respectively during the testing (validation) step. Also, a visual description through the contour diagram confirmed the capability of the recently proposed models to describe the physical variations of WRE under various operating conditions. Ultimately, a sensitivity analysis based on the MLP model was undertaken to shed light on the order of significance of operational factors in controlling WRE. Overall, the findings of the current research, in turn, have a satisfactory contribution to the efficient design of the water removal process from crude oil based on demulsifiers.

## Introduction

Oil emulsions can be categorized into two groups, i.e., simple and complex (multiple) emulsions, according to the water-to-oil ratio. The first group includes the water-in-oil (W/O) and oil-in-water (O/W), while the latter covers water-in-oil-in-water (W/O/W) and oil-in-water-in-oil (O/W/O). The phases volume ratio is the most fundamental factor in determining emulsion type. When the foregoing ratio tends to negligible or very large values, the phase with lower volume is recognized as the dispersed phase. The water-in-oil emulsion is the most common type of emulsion that is formed in different stages of crude oil extraction and refining^1–5^. The lack of proper separation processes for the mentioned emulsions makes negative impacts, not only on the quality and quantity of the resulting crude oil, but also on the equipment used in relevant industries. Increasing the costs required for storage and pumping, damaging transport systems and causing equipment corrosion are well-known examples regarding the harmful effects of the water presence in crude oil^6,7^. In order to resolve these problems, chemical, biological, mechanical, thermal, electrical and magnetic techniques^5^ are implemented to remove water from crude oil. In mechanical methods, the equipment installed on offshore platforms are costly, and take up a lot of space^1^. Although electrical techniques consume lower energy compared to the other methods, they cannot be assumed as efficient ways for the water removal process, due to the formation of small secondary droplets that need to be separated^5^. Applying heat on water-in-oil emulsions is associated with some drawbacks, such as equipment corrosion, and high economic cost, loss of light oil fractions and formation of air bubbles. This is why the thermal method is useful just when it is implemented along with the other methods. While biological demulsification is an eco-friendly and cost-effective method, it is time-consuming and sensitive to operating conditions^5^. Chemical water removal with demulsifiers is regarded as a widely used technique in the petroleum industries due to its simplicity, low heating and settling time and reasonable cost. The chemical demulsifiers employed in this method benefit from high economic efficiency and low toxicity. Since the aforementioned substances are expected to remove water from crude oil^1,5^, evaluation of factors affecting their performance is of particular importance to reach the optimal operating conditions.

The demulsification process is undertaken in three different steps, including flocculation, coalescence and sedimentation. Since the presence of natural surfactants (asphaltenes, naphthenic acids, waxes, etc.) in crude oil may enhance the stability of water-in-oil emulsion by the adsorption at the water–oil interface, demulsifiers should be able to overcome this stability^8,9^. In flocculation, the van der Waals force between water droplets is weakened and the droplets become clumped together. During coalescence, the interface film between the oil and water phases is destroyed and bigger droplets can be formed. The flocculation and coalescence steps can be accelerated at high temperatures and low oil viscosities^6^.

The demulsification efficiency strongly depends on the operating conditions, such as settling time, temperature, demulsifier concentration, the ratio of two phases, oil characteristics, etc.^6^. Mahdi et al.^9^ studied the influences of demulsifier concentration, temperature, wash water dilution ratio, settling time and mixing time salt removal efficiency (SRE) and water removal efficiency (WRE). Additionally, the fractional factorial design was applied to correlate SRE and WRE with operational factors. The proposed models showed satisfactory agreements with experimental findings. Adeyanju and Oyekunle^10^ investigated the performances of six diverse demulsifiers for water removal from two Nigerian crude oils. After determining the efficiency of each demulsifier in the water removal process, some demulsifier blends were prepared and the sample with the best performance was introduced.

The response surface method (RSM) and molecular simulations have been widely employed to assess the effect of operational factors on the crude oil demulsification^9,11–16^. Ahmadi et al. studied the impacts of oil to kerosene ratio, space velocity, temperature, demulsifier concentration and wash water ratio on the SRE and WRE of crude oil in an electrostatic desalting pilot plant based on the RSM approach^11^. According to the results, the oil to kerosene ratio was found as the most effective factor. Abdulredha et al.^12^ investigated the efficiency of demulsifier under various operating conditions. According to the analysis carried out based on the RSM method, the demulsifier dose, temperature and time were recognized the dominant factors for breaking the oil emulsion. Li and Chakraborty^8^ compared two RSM-based techniques, including Central Composite Design (CCD) and Box-Behnken design (BBD) to model the demulsification efficiency as a function of time, temperature, oil fraction and demulsifier concentration. It was found that the CCD technique performs much better than BBD in predicting the efficiency. In another work, the RSM technique was utilized by Azizi and Bashipour^13^ to model the demulsifier performance in the presence of Fe_2_O_3_ nanoparticles. Defining five input variables, including temperature, pH, water content, dose of nanoparticle and demulsifier concentration in the foregoing model resulted in the accurate prediction for removal efficiency. Wei et al.^17^ presented a molecular dynamic simulation to investigate the interfacial interaction between graphene oxide, as a demulsifier with different surface charge densities, and asphaltene molecules included in crude oil. This simulation was carried out to clarify the demulsification mechanism.

As opposite to conventional modeling approaches such as RSM, the use of intelligent methods in modeling is associated with many advantages, including high accuracy, simplicity and reliability^18,19^. Among all intelligent approaches, the multilayer perceptron (MLP) and gaussian process regression (GPR) have been widely used to solve petroleum engineering problems^20^. The MLP networks benefit from flexible structure and nonlinear activation functions that allows achieving high precisions in the prediction of complicated physical behaviors. On the other hand, the probabilistic framework of the GPR approach gives the opportunity to catch the uncertainties and develop extremely reliable predictive tools even based on limited number of data. Nabipour et al.^21^ yielded much accurate results in predicting the biofuel density based on the MLP network. Bagheri Vanani et al.^22^ presented an intelligent model for calculating the asphaltene content of crude oil based on the MLP method. The model showed an average relative deviation of 7.42% for 300 experimental data. Hashemi Fath et al.^23^ estimated gas-oil ratio of crude oil through the MLP network, and yielded an relative error of 14.90%. Mahdaviara et al.^24^ successfully implemented the GPR approach with diverse kernel functions for estimating the permeability in carbonate reservoirs. It was found that the mentioned method gives the R^2^ values exceeding 98% in estimating the permeability. In another work, Lv et al.^19^ derived reliable predictive tools for the diffusion coefficient of CO_2_ in bitumen and crude oils, and observed good consistencies between the model outcomes and experimental data.

According to the above literature survey, studies concerning the water removal from crude oil using demulsifiers are scarce. Additionally, there is still opportunity to develop reliable predictive tools for the foregoing under-researched process by directing focus towards the machine learning methods. Furthermore, it is vital to determine the most significant factors in controlling WRE from crude oil. Consequently, this study evaluates the performance of a commercial demulsifier, i.e., RP968Q for removing water from water-in-oil emulsions. The influences of temperature, demulsifier concentration and settling time on WRE are experimentally studied. Then, two machine learning based algorithms, including MLP and GPR are employed to design reliable predictive models for WRE. The validity and truthfulness of the proposed models are explored based on statistical indices and graphical descriptions, and the best predictive tool is specified. Ultimately, the most effective operational factors on WRE are determined based on a sensitivity analysis.

## Materials and methods

### Experimental procedure

#### Materials

In this study, a commercial demulsifier (RP968Q) from Baker Hughes Company, the united kingdom, was used for demulsification experiments. The physical and chemical properties of the demulsifier are shown in Table 1^25^. Hydrophobic demulsifiers are suitable for the demulsification of W/O emulsions^6^. So immiscible with water demulsifier was chosen for the demulsification of crude oil in this study. The crude oil used in this study was prepared from Dehloran oilfield, Ilam, Iran.

**Table 1:** Physical and chemical properties of the demulsifier^25^.

Physical state	Liquid
Color	Dark Brown
Odor	Aromatic
pH	5 to 8
Melting Point/Freezing point	< − 35 °C or < − 31 F
Flammability (solid, gas)	May be combustible at high temperature
Flash point	Closed cup: > 60 °C (> 140 F)
Relative density	0.917 to 0.987 at 16 °C
Solubility	Immiscible with water, Soluble in Aromatic solvents

#### Demulsification process

The experiments for determining WRE were conducted based on the bottle-test procedure. The experimental setup consists of water bath with a digital thermostat, graduated cylinders with a screw cap, and micropipette. After obtaining constant temperature in a water bath, the bottle tests were performed with several cylinders contain 50 ml of crude oil at a specified temperature. The different amounts of demulsifier were, in turn, injected into each of the 50 ml of the crude oil according to the specific volumes using micropipette. After tightening the screw cap, the graduated cylinders were shaken to ensure the complete mixing of the demulsifier and the crude oil. The separation of the water from the crude oil was recorded at different times by measuring the interface between the oil and the separated water that settled at the bottom^10,12^. Different amounts of demulsifiers were added to a series of an equal amount of crude oil (50 ml) in a graduated cylinder using a micropipette. The demulsification experiments were carried out in different operation conditions. Table 2 represents the ranges and levels of demulsifier concentration, temperature and settling time analyzed in this study.

**Table 2:** The operating conditions of the WRE tests carried out in this study.

Parameter	Range evaluated	Levels evaluated
Demulsifier Concentration (ppm)	10–60	10, 20, 30, 40, 50, 60
Temperature (°C)	25–70	25, 35, 45, 55, 65, 70
Settling time (min)	0.5–30	0.5, 1, 2, 3, 5, 10, 15, 30

The water removal efficiency (WRE) can be calculated based on the volume of separated water after adding the demulsifier (V_t_) and the original volume of water (V_0_) in crude oil^10,12^:

1 $$WRE \left(\%\right)=\frac{{V}_{t}}{{V}_{0}}\times 100$$

### Machine learning algorithms

#### Multilayer perceptron (MLP)

**Figure 1:**
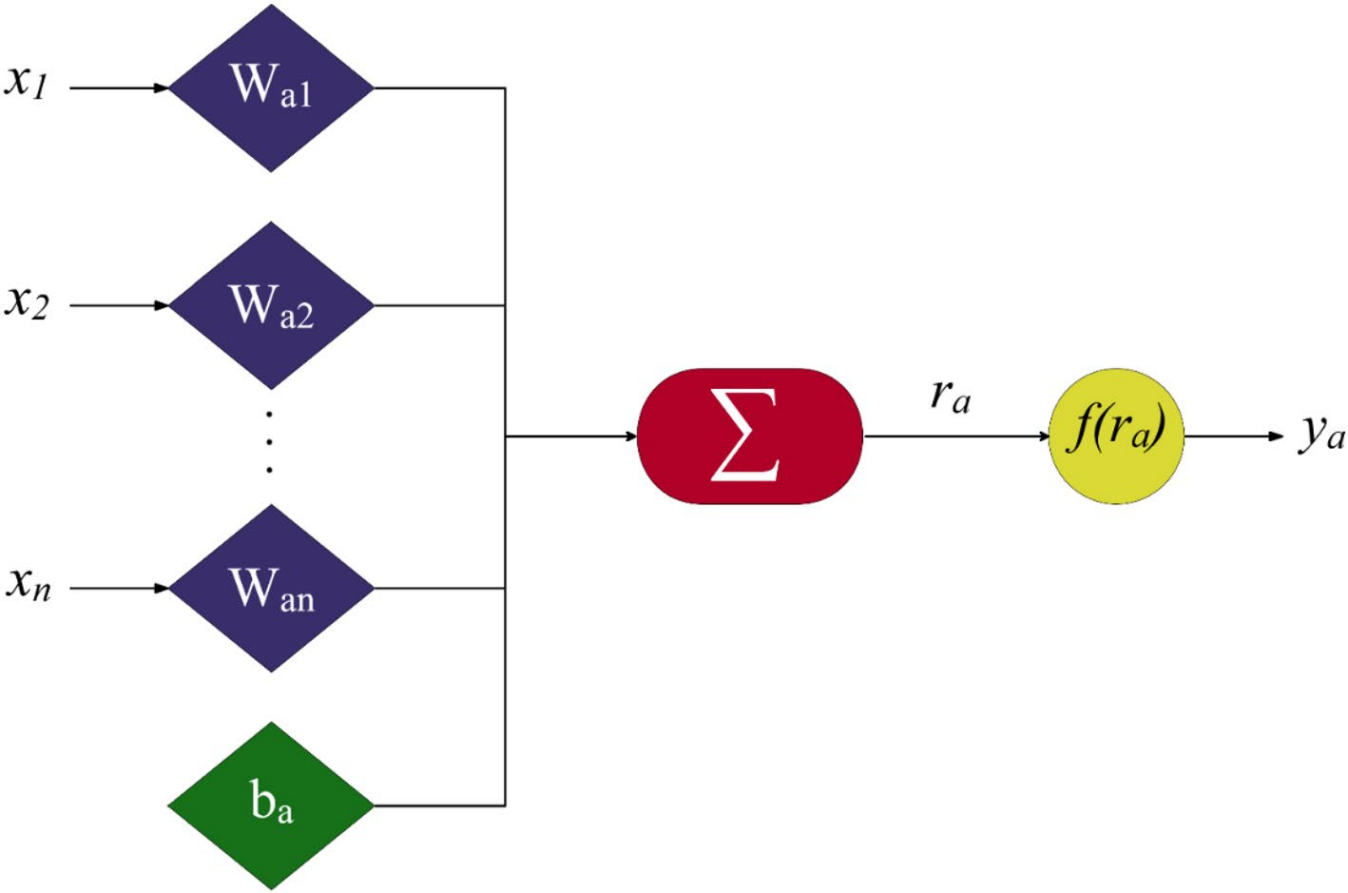
The details of an artificial neuron.

The capable machine learning approach of MLP follows a process similar to that observed in the nervous system of humans, and it is mainly implemented to solve complicated mathematical problems, including approximation, classification and pattern recognition. This is done through a parallel algorithm, in which a set of data is utilized to train the network, and the artificial neurons are responsible for transferring the information. Figure 1 shows the structure of an artificial neuron included in the MLP network. This neuron is described as the following mathematical forms:

2 $${r}_{a}=\sum_{i=1}^{n}{x}_{i}{W}_{ai}+{b}_{a}$$

3 $${y}_{a}=f\left({r}_{a}\right)$$

where ${r}_{a}$, ${x}_{i}$, ${W}_{ai}$, ${b}_{a,}$ and ${y}_{a}$ stand for linear combiner, *i*th input factor, synaptic weight, neuron bias, and activation function, respectively.

**Figure 2:**
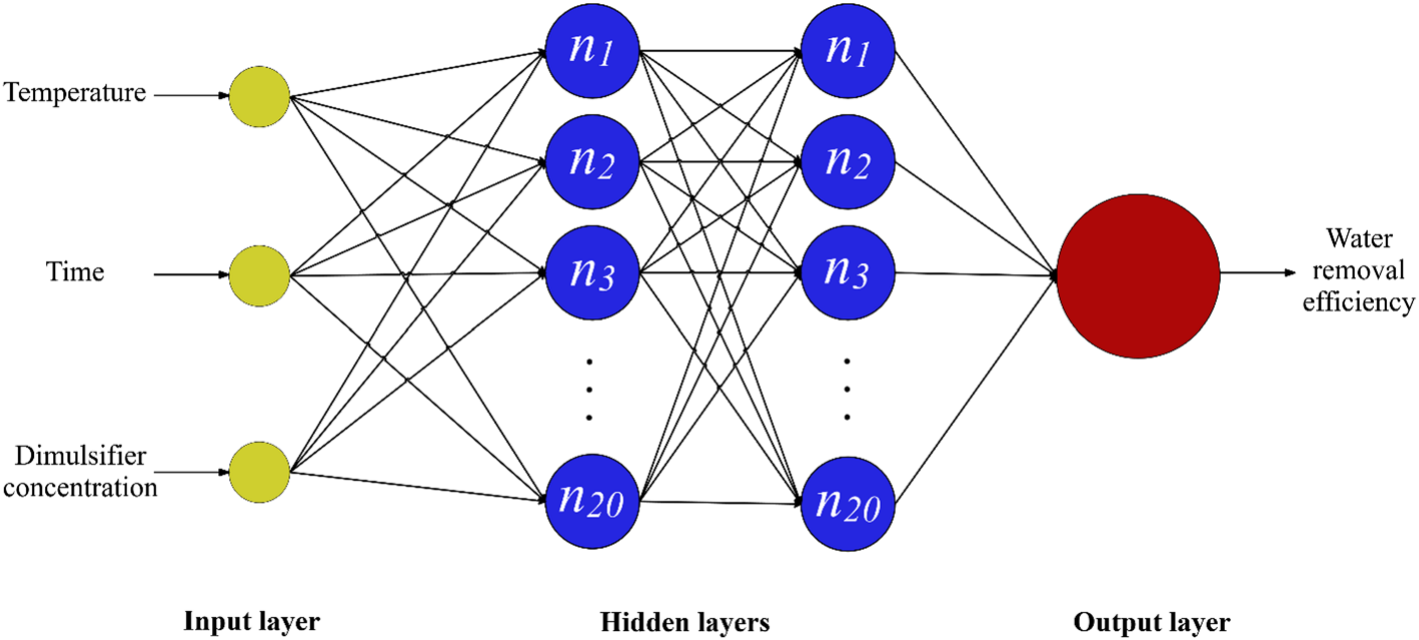
The MLP network designed to model the water removal efficiency.

MLP is recognized as feed-forward network, i.e. it processes the information only in one direction. The graphical description of the MLP network designed to model WRE has been illustrated in Fig. 2. It is clear that it includes three linked layers, containing some artificial neurons. The input, hidden and output layers are responsible for introducing the information to the network, specifying the network parameters and displaying the outcomes, respectively. It should be emphasized that the architecture of hidden layer depends on the complexity of problems, and may include some independent layers with different numbers of neurons in each of them. However, the number of neurons in the first and last layers equal to the number of input and output variables, respectively. The MLP network detects the system nonlinearity via the activation functions included in the hidden layer neurons. In this study, after trying a variety of structures, a double hidden layer network, including 20 neurons in each of them, exhibited the best performances for modeling WRE from crude oil. Moreover, the neurons benefit from the tan-sigmoid function as activation function.

Optimizing the values of hypermeters, such as weights and biases is a vital stage in the design of MLP models, since they considerably affects the capability of the network. For this purpose, the back propagation (BP) algorithm specifies the foregoing parameters, so that the minimum value of deviation function is obtained:

4 $$D={\left({y}_{pre}-{y}_{exp}\right)}^{2}$$

After applying each training data point, the value of deviation function is spread in the network, and the values of weights and biases are re-adjusted using the BP algorithm. Herein, the Bayesian Regularization approach has been employed to train the BP algorithm.

#### Gaussian process regression (GPR)

Recently, due to robustness and capabilities of non-parametric machine learning approaches, they have received many attentions for solving engineering problems. Among the foregoing approaches, GPR is a widely used machine learning algorithm, by which a gaussian joint probability distribution is provided. The main advantages of GPR are high accuracy and ability to modulate the hyperparameters. This method is also capable to catch the uncertainty of analyzed samples.

The GPR-based learning process is accomplished through a probabilistic framework, in which a training dataset $T=\left[\left({x}_{i},{y}_{i}\right) i=\mathrm{1,2},\dots ,n\right]$ is provided. It should be noted that ${x}_{i}$ and ${y}_{i}$ stand for the input variables vector and target function, respectively. Thus, the predictive model provides the output function distribution in any point through the following approximation:

5 $${y}_{i}=L\left({x}_{i}\right)+{\varepsilon }_{i}$$

where $L\left({x}_{i}\right)$ is the latent function corresponding to the input variables (${x}_{i}$), and its values constitutes a random variable. Furthermore, ${\varepsilon }_{i}$ represents the gaussian noise, which its mean and variance are 0 and ${\sigma }_{n}^{2}$, respectively:

6 $${\varepsilon }_{i}=N(0,{\sigma }_{n}^{2})$$

Consequently, the target function can be easily approximated by defining a mean function $m(x)$ and a covariance function $cov(x,{x}{\prime})$. The predictive probability distribution for the input variables, ${x}^{*}$ can be defined as:

7 $${\widehat{y}}^{*}=m\left({x}^{*}\right)+{k}_{*}^{T}{\left(K+{\sigma }_{n}^{2}I\right)}^{-1}\left(y-m\left({x}^{*}\right)\right)$$

8 $${\sigma }_{{y}^{*}}^{2}={k}_{*}+{\sigma }_{n}^{2}-{k}_{*}^{T}{\left(K+{\sigma }_{n}^{2}I\right)}^{-1}{k}_{*}$$

where ${k}_{*}$ may be defined as ${\left[{k}_{*}\right]}_{i}=cov({x}_{i},{x}^{*})$, $K$ stands for a covariance matrix, which its elements are ${\left[K\right]}_{i,j}=cov({x}_{i},{x}_{j})$, and $I$ shows the identity matrix.

As the predictive probability distribution is specified by the hyperparameters, an optimization process should be performed to determine these factors. In the GPR approach, the log-likelihood function is maximized during the training stage in order to calculate the hyperparameters:

9 $${\text{log}}p\left(y|X\right)=-\frac{1}{2}{y}^{T}{\left(K+{\sigma }_{n}^{2}I\right)}^{-1}y-\frac{1}{2}{\text{log}}\left(\left|\left(K+{\sigma }_{n}^{2}I\right)\right|\right)-\frac{n}{2}{\text{log}}(2\pi )$$

where *n* represents the number of training data points.

### Error analysis

To evaluate the capability and exactness of the proposed models for estimating WRE, the average absolute relative error (AARE), relative root mean squared error (RRMSE) and standard deviation (SD) were calculated^26^:

10 $$AARE (\%)=\frac{\sum \left|{E}_{i}\right|}{N}\times 100$$

11 $$SD \left(\%\right)=\sqrt{\frac{\sum {\left({E}_{i}-\overline{{E}_{i}}\right)}^{2}}{N-1}}\times 100$$

12 $$RRMSE (\%)=\frac{\sqrt{\frac{1}{N}\sum {\left({WRE}_{pre}-{WRE}_{exp}\right)}^{2}}}{\frac{1}{N}\sum {WRE}_{exp}}\times 100$$

where ${E}_{i}$ stands for the relative error of *i*th estimated data,

13 $${E}_{i}=\frac{{WRE}_{pre,i}-{WRE}_{exp,i}}{{WRE}_{exp,i}}$$

## Results and discussion

### Experimental determination of the water removal efficiency from crude oil

#### Influences of time and demulsifier concentration

**Figure 3:**
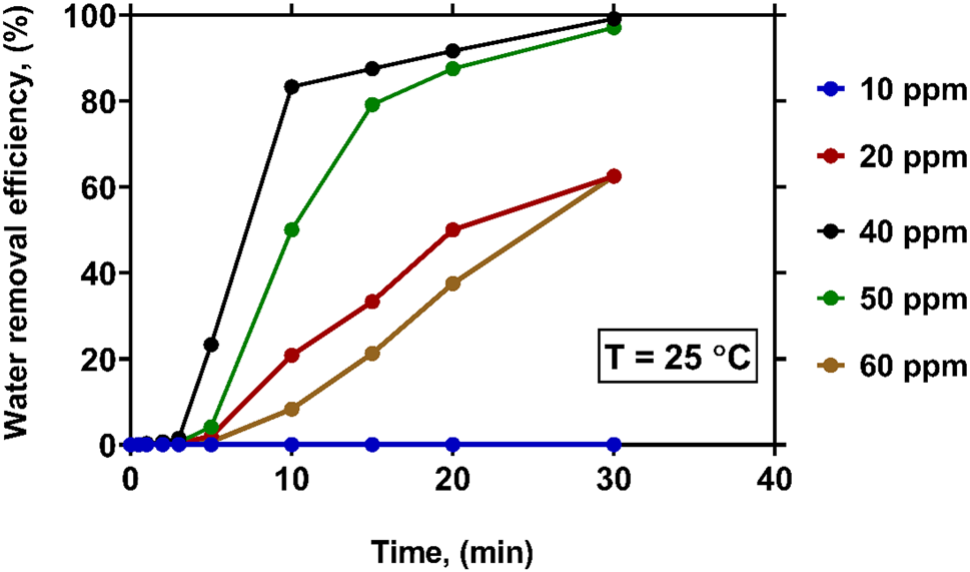
The effect of demulsifier concentration on WRE at temperature of 25 $\mathrm{^\circ{\rm C} }$.

Figure 3 represents the variations of WRE with time at various levels of demulsifier concentration under a constant temperature of 25 °C. As can be seen, a low concentration of demulsifier (10 ppm) is not enough to break the W/O emulsion, so that the phases remain unseparated. This can be explained by the presence of elastic and viscous film around the water droplets under this condition^27^. By increasing the demulsifier concentration up to 40 ppm, the WRE from crude oil at the initial moments is gradually increased. After that, it is dramatically enhanced over time at any concentration. When the demulsifier concentration is 40 ppm, a WRE of 23% is obtained after 5 min. The foregoing value reaches more than 99% after 30 min. A high concentration of demulsifier may increase the rate of coalescence of the water droplets due to the thinning of the interfacial film^27^. The size of water droplets is increased by sticking the droplets (coalescence), which results in the reduction of the water-in-oil emulsion stability^28^. Since the demulsifier used in this study (RP968Q) is immiscible with water, it migrates to the two-phase interface during mixing with crude oil. In fact, demulsifiers have interfacial tension gradient that arises from the difference between interfacial tension inside and outside of the film. Due to the high surface activity of demulsifiers, they can invert the interfacial tension gradient after mixing with crude oil. Hence, the coalescence of water droplets can be occurred^29^.

The demulsifier concentrations exceeding 40 ppm not only make no positive impacts on WRE, but also cause it to decrease. As shown in Fig. 3, the WRE values obtained at 60 ppm concentration are even less than those obtained at 20 ppm. Accordingly, the overdose of the demulsifier only leads to a higher economic cost. The adsorption rate at the interface of water drops is directly dependent to the demulsifier concentration. In fact, the adsorption of demulsifier at the interfacial layer can prevent the emulsification phenomenon. Afterward, the interfacial tension between oil and water is decreased, which contributes to the enhancement of the coalescence process^4,12,28^. In other words, the demulsifier injection affect the dynamic properties of the oil–water interface^29^. During the overdose of demulsifier, the orientation of the hydrophobic part of the demulsifier molecules are changed, and this gives the demulsifier opportunity to bond with the rest of the water droplets in the opposite direction^29^. In fact, at very high concentration (overdose), the demulsifier act as an emulsifier agent, which enhances the stability of crude oil emulsion^12^.

While increasing time contributes to the gravitational settling, and enhances the demulsifier diffusion through the interface^6^, it can also increase operational costs. Additionally, a high settling time may be associated with the re-emulsification process. This is why determining the optimal settling time is crucial. The settling time can be minimized by adjusting temperature and demulsifier concentration.

**Figure 4:**
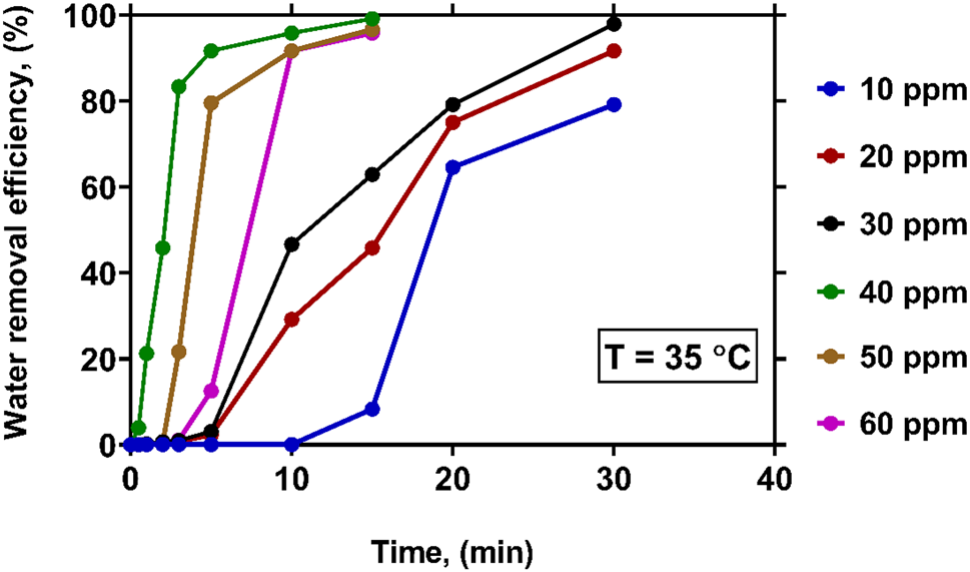
The effect of demulsifier concentration on WRE at temperature of 35 $\mathrm{^\circ{\rm C} }$.

The variations of WRE with time at various levels of demulsifier concentration under a constant temperature of 35 °C have been depicted in Fig. 4. As it is evident, by increasing temperature, higher WRE values can be obtained even at very low demulsifier concentrations. This can be explained by the reduction of viscosity and interfacial surface tension at higher temperatures^12^. When the demulsifier concentration is 10 ppm, the WRE values of 8% and 79% are observed after 15 and 30 min, respectively. As the concentration of the demulsifier is increased up to 40 ppm, a noticeable improvement is made in WRE. At the concentrations of 10, 20, 30 and 40 ppm, the WRE from crude oil reaches 8.33%, 45.83%, 62.92% and 99.17%, respectively. This observations highlight the fact that at the concentration of 40 ppm, a higher WRE value can be obtained in a short settling time. Similar to those observed under the temperature of 25 °C, a further increase in the concentration of demulsifier (overdose) is associated with the reduction of WRE, and the WRE curves pertinent to the concentrations of 50 and 60 ppm lie below that of 40 ppm. Hence, determining the optimal concentration of demulsifier is of special importance in the water removal process from crude oil.

**Figure 5:**
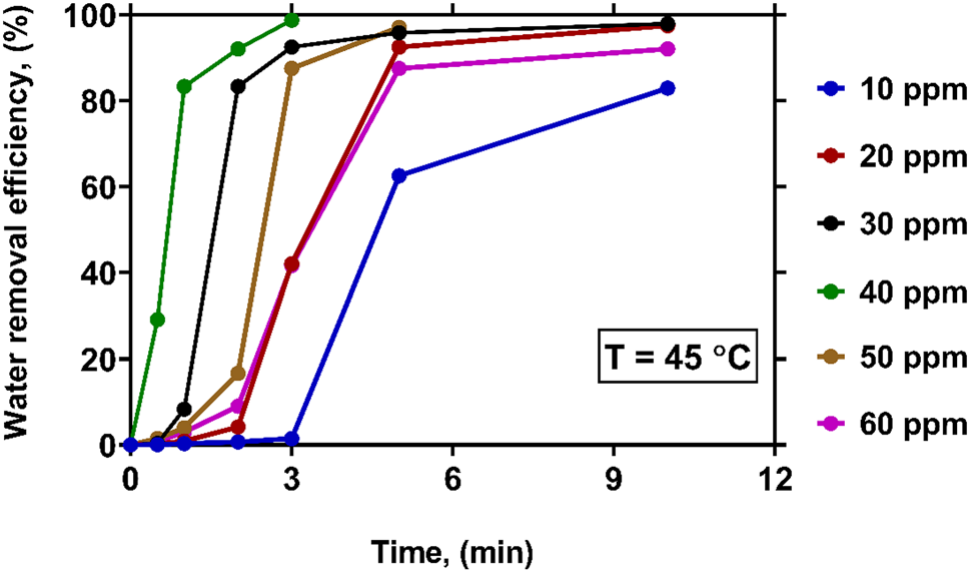
The effect of demulsifier concentration on WRE at temperature of 45 $\mathrm{^\circ{\rm C} }$.

Figure 5 illustrates the variations of WRE with time at various levels of demulsifier concentration under a constant temperature of 45 °C. A glance at the figure indicates that at all demulsifier concentrations analyzed in this study, more than 80% of water is removed in the first 10 min of the demulsification process. Comparing these values with those obtained under 25 $^\circ{\rm C}$ and 35 °C demonstrates the significance of temperature in controlling WRE from crude oil. The time required to reach the maximum value of WRE (> 99%) at 25 °C and 35 °C were found to be 30 and 15 min, respectively. While, the mentioned time is reduced to 3 min at 45 °C. The significant improvement of WRE with raising temperature can be justified based on the following arguments^6,12,27^:

1. The difference between the polarities and densities of water and oil is elevated at high temperatures due to the reduction of oil viscosity.
2. The thermal energy of water droplets is boosted by raising temperature, which leads to an increase in the coalescent process.
3. The interfacial film between two phases and the chemical equilibrium is considerably affected by temperature. Furthermore, the formation of the interfacial film is weakened at high temperatures, and the solubility of emulsifiers in oil is increased.

**Figure 6:**
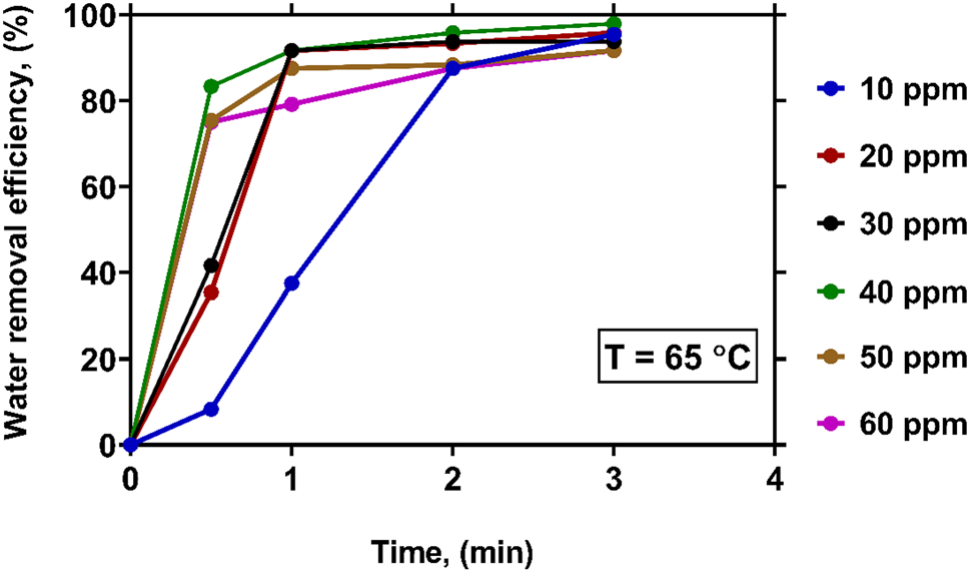
The effect of demulsifier concentration on WRE at temperature of 65 $\mathrm{^\circ{\rm C} }$.

The variations of WRE with time at various levels of demulsifier concentration under a constant temperature of 65 °C have been described in Fig. 6. As it is observed, WRE is sharply enhanced in a short period of time under the foregoing temperature. At a very low demulsifier concentration, i.e., 10 ppm, it takes just 3 min to reach a WRE exceeding 95%. By increasing the concentration of demulsifier up to 40 ppm, a noticeable improvement is made in the WRE value measured at the initial moments. For instance, at 40 ppm concentration, the WRE value reaches around 83% after just 30 s. It is seen that the negative effect of the demulsifier overdose on WRE is also observed at 65 °C. Meanwhile, the concentration dependence of WRE is diminished at high temperatures. The governing mechanisms of the demulsification process at high temperature are Brownian motion and mass transfer across the interface^6,27^. Thus, the coalescence of the water droplets is increased, and the separation of water from crude oil is occurred due to their different density and polarity.

**Figure 7:**
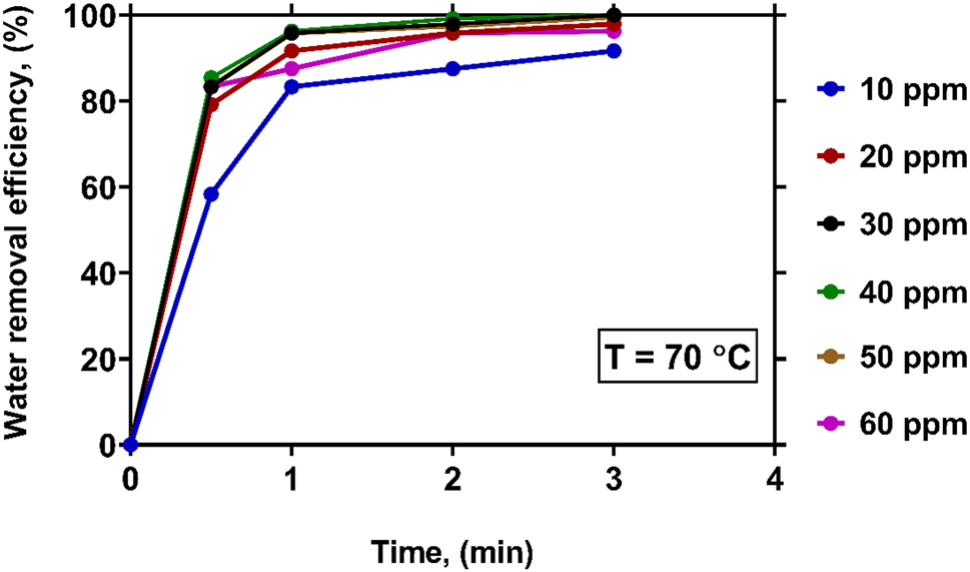
The effect of demulsifier concentration on WRE at temperature of 70 $\mathrm{^\circ{\rm C} }$.

Figure 7 sketches the variations of WRE with time at various levels of demulsifier concentration under a constant temperature of 70 °C. It is seen that a large fraction of water can be promptly removed using a demulsifier of any concentration. The WRE values obtained at all concentrations exceed 82% in a maximum of 3 min. At the concentrations of 30, 40 and 50 ppm, a complete water removal (WRE of 100%) is achieved. In fact, increasing temperature leads to a viscosity reduction in the crude oil, and enhances the breakdown of the water in oil emulsion^27^.

Overall, increasing the temperature makes a significant positive impact on the performance of the demulsifier, and makes it possible to separate water from crude oil at low concentrations or even without demulsifier. However, performing the demulsification process at temperatures higher than the optimal value cannot be assumed as an efficient way to improve WRE, since it causes energy losses and higher operating costs. Additionally, the components with low boiling point have the opportunity to evaporate at temperatures above 70 °C^28^. On the other hand, a high level of temperature causes the water droplets to slip, and restricts their binding^29,30^. These bubbles can reduce the apparent viscosity of water drops by adhering to their surfaces. Thus, they cannot be separated from crude oil by the sedimentation process^29^.

#### Influence of temperature

**Figure 8:**
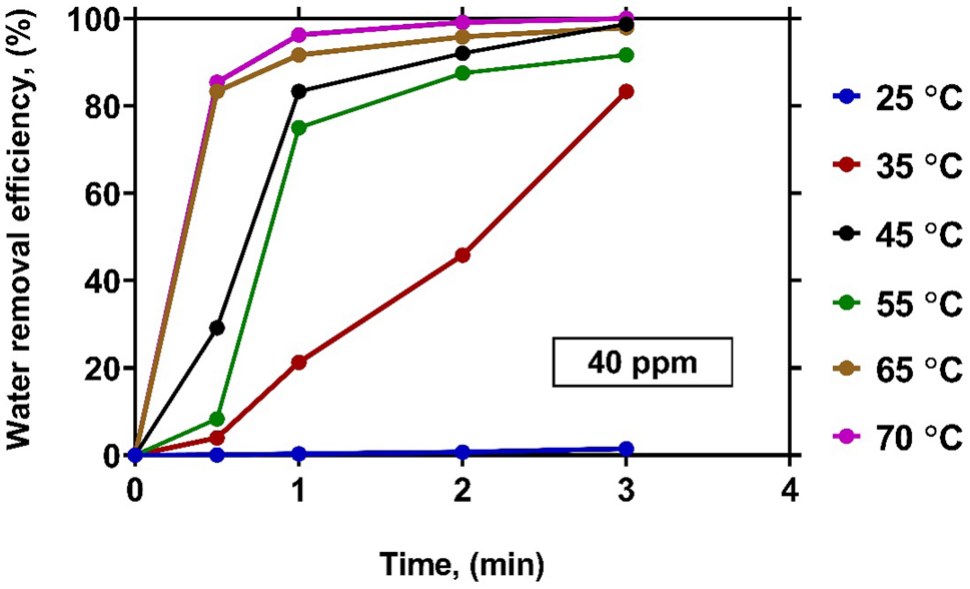
The effect of temperature on WRE at demulsifier concentration of 40 ${\text{ppm}}$.

In order to show the exact effect of temperature, the variations of WRE with time at various levels of temperature under a constant concentration of 40 ppm have been illustrated in Fig. 8. As it is observed, although the concentration of 40 ppm is the optimal amount of demulsifier to achieve the highest WRE, the performance is greatly affected by temperature. At ambient temperature, almost no progress is observed in the water removal within 3 min. However, WRE is significantly enhanced by increasing temperature. At temperatures higher than 45 °C, WRE reaches approximately 100% after 3 min.

### Smart modeling of the water removal efficiency

#### Development of the machine learning-based models

In order to demonstrate the application of intelligent methods for describing the WRE from crude oil, 203 experimental data measured in this study were employed to design predictive models for WRE based on the MLP and GPR approaches. Firstly, around 80% of experimental samples, called training data, were used for establishing the intelligent models. Afterward, the capability of the developed models for predicting the unseen data was assessed by the remaining 20% of samples, called testing data. Table 3 compares the error metrics pertinent to the novel smart models to predict WRE during training and testing processes. As seen, both MLP and GPR models provide exact predictions with AAREs of 1.40% and 1.29%, RRMSEs of 0.01% and 0.02%, and SDs of 5.74% and 4.55%, respectively for total data. For the training dataset, the GPR model exhibits slightly better performances compared to the MLP model. However, the MLP model presents superior predictions for testing data with AARE, RRMSE, and SD values of 0.84%, 0.01%, and 3.58%, respectively. Hence, it can be found that while both smart models have excellent capabilities for predicting the WRE, the MLP model is slightly more reliable for this purpose due to its better results for test data. This observation lies in the fact that the flexible structure of the MLP network gives the opportunity to adjust the number of hidden layers, neurons and weights in order to reach desirable estimations. On the other hand, since the physical variations of the WRE from crude oil are nonlinear (as shown in last section), the activation functions included in the MLP network contribute to better predictions of the foregoing nonlinear trends. Overall, this analysis reveals that the machine learning algorithms are robust and capable tools to predict the WRE from crude oil at the ranges of settling time, temperature and demulsifier concentration summarized in Table 2. Hence, they can be implemented to derive more comprehensive models when further experimental data for diverse types of demulsifiers are available.

**Table 3:** Error metrics of the smart models for predicting the WRE from crude oil.

Dataset	Error metric	Values obtained by the GPR model	Values obtained by the MLP model
Training (162 data)	AARE, (%)	1.18	1.53
	RRMSE, (%)	0.01	0.02
	SD, (%)	4.31	6.16
Testing (41 data)	AARE, (%)	1.65	0.84
	RRMSE, (%)	0.01	0.02
	SD, (%)	5.30	3.58
Overall (263 data)	AARE, (%)	1.29	1.4
	RRMSE, (%)	0.01	0.02
	SD, (%)	4.55	5.74

**Figure 9:**
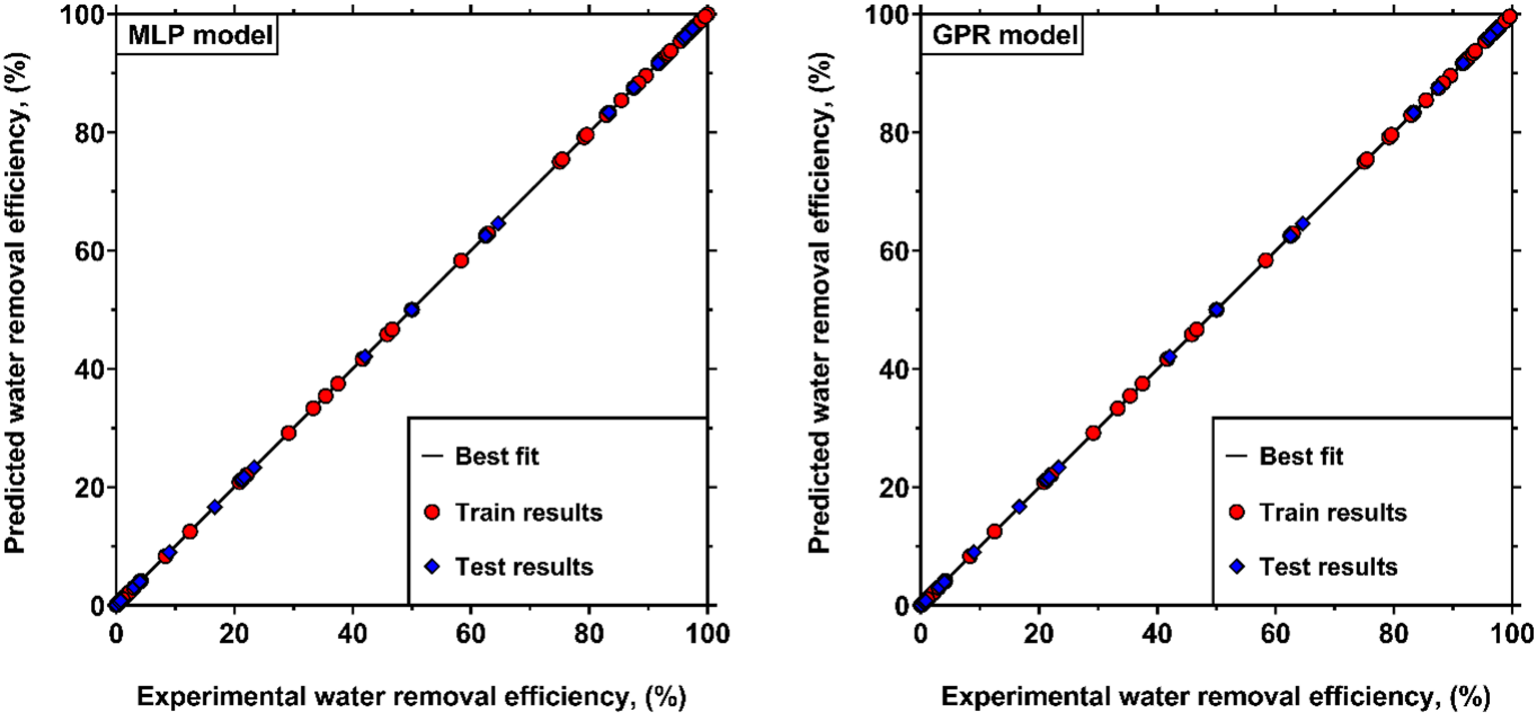
Comparison between experimental values of WRE and the outcomes of the newly developed smart approaches.

Figure 9 depicts the WRE values predicted by the smart models against the actual data. Obviously, the predictions of both MLP and GPR models for train and test datasets are much close to the best-fit line, representing the excellent capabilities of the machine learning algorithms to predict WRE. However, as discussed earlier, the MLP model can be recognized as the more reliable model, as it provides better results during the testing process.

#### Trend analysis of the novel models

In order to illustrate the prediction capabilities of the proposed models, in this section, a trend analysis is performed based on the outcomes of the MLP model as the most reliable predictive approach developed in this study.

**Figure 10:**
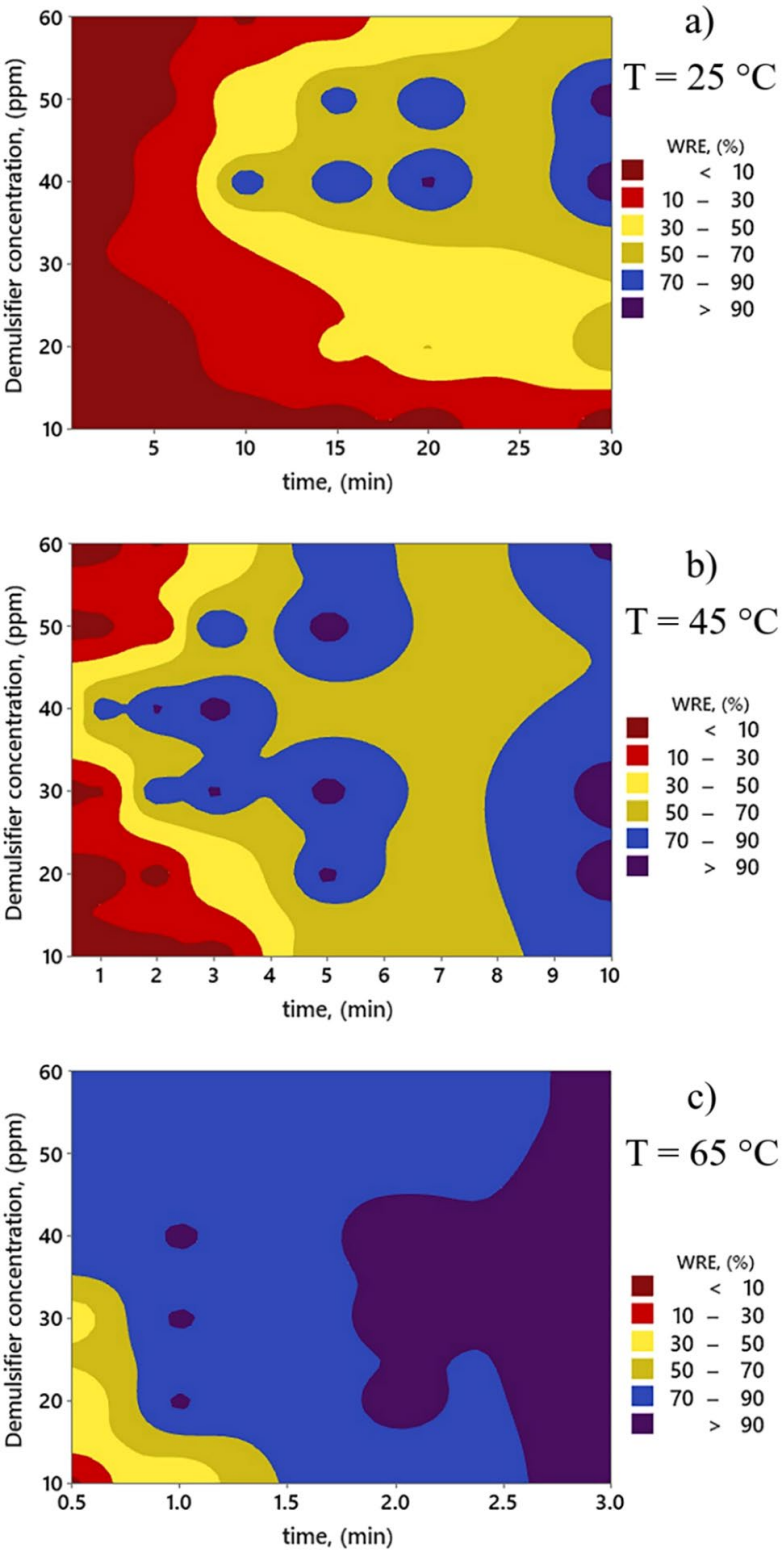
Contour plot pertinent to the WRE values predicted by the MLP model under various times, demulsifier concentrations and temperatures.

The variations of WRE with time and demulsifier concentration at the temperatures of 25 °C, 45 °C, and 65 °C based on the outcomes of the MLP model have been sketched in the contour plot of Fig. 10. This graphical approach visualizes the variation of WRE through a color spectrum, ranging from dark red to purple, which denote the WREs less than 10% to exceeding 90%, respectively. A glance at the figure implies the point that the red portions (low WREs) are more dominant at low temperatures, and they are gradually vanished with increasing temperature. This means that there is a direct correlation between WRE values and temperature. Additionally, at all temperatures, the blue areas with purple shadings (high WREs) are expanded over time. Hence, more removal efficiency can be obtained when the duration of the demulsification process is increased. On the other hand, the increase of demulsifier concentrations up to 40 ppm contributes to the enhancement of WRE. After that, an inverse relationship between demulsifier concentration and WRE is observed. As it is evident, the MLP model appropriately describes the alternations of WRE under different condition, and its outcomes are in accordance with the experimental findings presented in last section.

**Figure 11:**
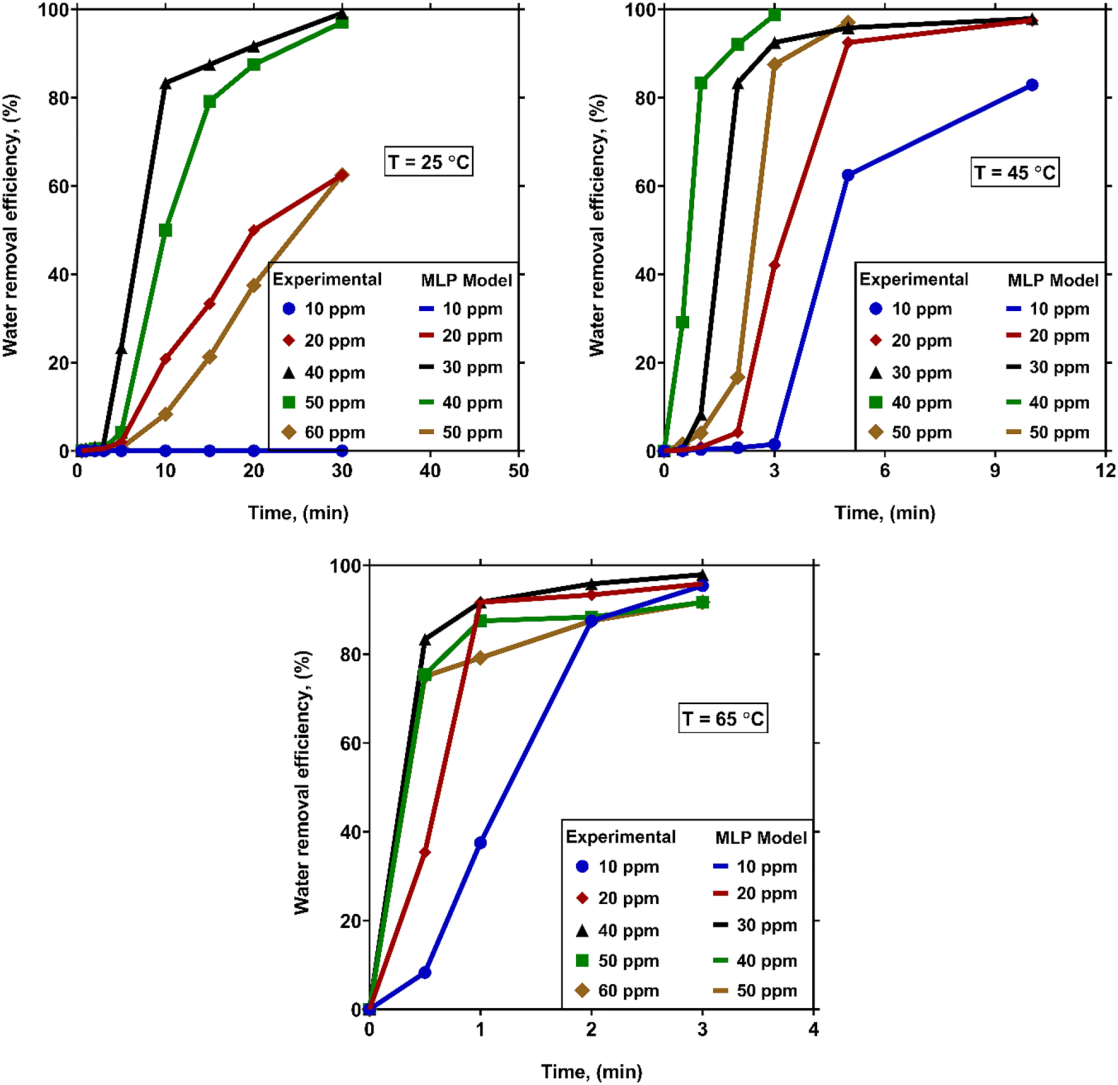
Comparison between the experimental values of WRE with those estimated by the MLP model at diverse settling times, temperatures and demulsifier concentrations.

For affirming the capability of the smart models to describe the physical variations of WRE versus operating factors, Fig. 11 provides a comparison between the values of WRE from crude oil obtained by the experimental measurements with those predicted by the MLP model at different settling times, temperatures and demulsifier concentrations. As it is obvious, the MLP model favorably describes the physical attitudes of WRE at all levels of operational factors. Moreover, its outcomes are in very nice agreement with the experimental findings. Consequently, the MLP model can be regarded as a prosperous and trustworthy predictive tool for predicting the physical trends of WRE.

### Sensitivity analysis

In order to optimize the demulsification process, identifying the most influential factors on WRE is of particular importance. To fulfill this need, the Pearson's correlation coefficient between WRE values calculated by the MLP model and the operating factor were determined. This coefficient can be calculated as follow between two given factors:

14 $$P\left({x}_{1},{x}_{2}\right)=\frac{\sum_{i=1}^{n}\left({x}_{1i}-\overline{{x}_{1i}}\right)\left({x}_{2i}-\overline{{x}_{2i}}\right)}{\sqrt{\sum_{i=1}^{n}{\left({x}_{1i}-\overline{{x}_{1i}}\right)}^{2}\sum_{i=1}^{n}{\left({x}_{2i}-\overline{{x}_{2i}}\right)}^{2}}}$$

The minimum and maximum values of $P\left({x}_{1},{x}_{2}\right)$ are − 1 and 1, which represent the highest levels of inverse and direct correlations between two factors, respectively. In contrast, when its value is close to zero, it can be found that the relationship between the factors is negligible.

**Figure 12:**
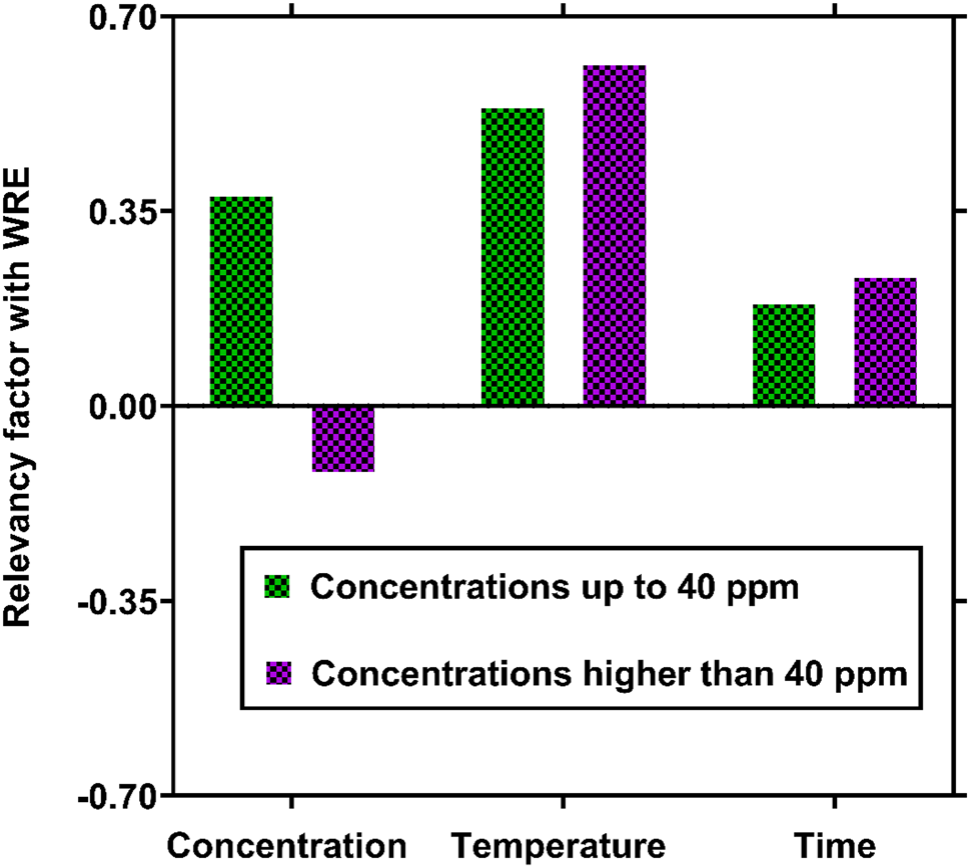
Relevancy factors between operating factors and the WRE calculated by the MLP model.

Figure 12 demonstrates the relevancy factor between WRE and time, temperature, and demulsifier concentration. It is seen that WRE has direct relationships with time and temperature. However, the demulsifier concentration exhibits contradictory behaviors at low and high temperatures. While it directly affects WRE at concentrations up to 40 ppm, there is an inverse relationship between this factor and WRE at higher concentrations. All these results are in line with those observed in experimental measurements, and this is another testimony regarding the capability of the intelligent methods for predicting WRE from crude oil. Figure 12 also shows that temperature is the most important factor in controlling WRE at low concentrations, which is followed by demulsifier concentration and time, respectively. In contrast, at high concentrations, the most effective factors are temperature, time, and demulsifier concentration, in decreasing order. Accordingly, it can be concluded that increasing temperature is the best way to make a significant improvement in WRE.

## Concluding remarks

This study aimed at investigating the water removal efficiency from crude oil using a commercial demulsifier. The variations of WRE with temperature, demulsifier concentration, and time were experimentally studied. The experimental data were also employed to design predictive models for WRE based on the machine learning algorithms of GPR and MLP. The main findings of this research can be expressed as follows,

- WRE from crude oil was strongly dependent on temperature and experienced an ascending trend with the growth of temperature. Furthermore, the change of temperature can also affect the correlation of WRE with the other operational factors.
- Increasing the demulsifier concentration up to 40 ppm led to an increase in WRE. However, a higher concentration did not make a positive impact on WRE. In addition, the concentration dependence of WRE was diminished at high levels temperatures.
- WRE was dramatically enhanced over time, and this fact was more obvious at higher temperatures and demulsifier concentrations.
- After defining three variables, including time, temperature and demulsifier concentration, two intelligent models were proposed to predict WRE from crude oil. Both MLP and GPR models were found as reliable predictive tools for the scope of this study. However, the MLP model presented more reliable predictions for the testing (validation) dataset with AARE, RRMSE, and SD values of 0.84%, 0.01%, and 3.58%, respectively.
- The proposed models also performed excellently in predicting the physical trends of WRE under different operating conditions.
- A sensitivity analysis was undertaken based on the newly developed models. The results were entirely in line with those observed in experimental investigations. It was found that temperature is the most fundamental factor in controlling crude oil demulsification.

Consequently, the present study shed light on the influences of settling time, temperature and demulsifier concentration on WRE from crude oil, and demonstrated the application of the intelligent methods, i.e. MLP and GPR for predicting the mentioned influences. The results obtained can contribute to the optimal design of the water removal process from crude oil. However, further experimental investigations on the performances of other types of demulsifiers for water removal from crude oil are required to design more comprehensive intelligent models.

## Data Availability

All data generated during this study are included in this published article.
